# Histological and biological evaluation of wheat straw–derived nanocellulose in a rat model of cutaneous wound healing

**DOI:** 10.3389/fbioe.2026.1800416

**Published:** 2026-04-01

**Authors:** Nahidah Ibrahim Hammadi, Ahmed J. Alfahdawi, Athraa Basheer Radhi, Alaa Imad Abdulrazzaq

**Affiliations:** 1 Department of Laboratory and Clinical Sciences, College of Pharmacy, University of Anbar, Anbar, Iraq; 2 Department of Pathological Analysis, College of Applied Science, University of Fallujah, Fallujah, Iraq

**Keywords:** angiogenesis, biomaterials, cutaneous wound healing, fibroblast migration, histology, nanocellulose, wheat straw

## Abstract

**Background:**

Healing of cutaneous wound is a complex biological process that requires the migration of fibroblasts, production of extracellular matrix (ECM) component, angiogenesis and a highly regulated expression pattern of inflammatory mediators. Owing to the interest in wound dressings based on renewable biomaterials, much attention has been paid to their biocompatibility and sustainability. Within these materials, nanocellulose features the physical and chemical properties to promote important cellular processes for tissue regeneration.

**Methods:**

Wheat straw–derived nanocellulose was isolated and its nanostructure and physicochemical properties were characterised using FESEM, XRD, EDX, and FTIR analyses. *In vitro*, its biological activity was evaluated in NIH-3T3 fibroblasts through cell viability, migration, and expression of wound-healing–related genes and proteins (*n* = 3). The effects of nanocellulose on wound healing were further evaluated using a rat full-thickness skin wound model (*n* = 10 per group), with macroscopic and histological assessments conducted at 3, 7, and 14 days after wounding.

**Results:**

The extracted nanocellulose exhibited a cellulose I crystalline structure and showed no cytotoxic effects on fibroblasts (p > 0.05). Nanocellulose-conditioned medium significantly enhanced fibroblast migration compared with control conditions (p < 0.001) and upregulated the expression of *Col1a1* (p = 0.0093), *Col3a1* (p = 0.0308), and *Vegfa* (p = 0.0175), accompanied by increased VEGF protein secretion (p = 0.0007) without concurrent elevation of inflammatory marker IL-6 (p > 0.05). In the rat wound model, nanocellulose treatment significantly accelerated wound closure at days 3, 7, and 14 (p < 0.01–0.0001) and improved histological features of healing, including reduced inflammatory cell infiltration, more organised granulation tissue formation, enhanced angiogenesis, and earlier re-epithelialisation.

**Conclusion:**

These results suggest that nanocellulose derived from wheat straw can promote cutaneous wound healing by stimulating fibroblast-mediated matrix synthesis and angiogenesis without resulting in excess inflammatory stimulation. Taken together, the *in vitro* and *in vivo* findings demonstrate that nanocellulose is not only a renewable biomaterial but also an active biological material for use as functional wound-healing dressings.

## Introduction

Wound healing is a complicated and highly orchestrated biological process that involves overlapping stages of inflammation, proliferation, and remodelling. After injury, inflammatory cells clear debris and pathogens from the wound bed, and fibroblasts migrate into the site of damage synthesising extracellular matrix (ECM) that defines a structural context to facilitate new tissue formation and re-epithelialisation. In this context, the modulatory function of fibroblasts in collagen deposition, wound contraction and tissue remodeling is extensively reported in experimental and clinical studies (Cialdai et al., 2022; Bainbridge, 2013; Talbott et al., 2022).

A key component of the proliferative phase of the healing process, angiogenesis ensures the delivery of oxygen and nutrient requirements for the metabolism of fibroblasts and the deposition of the extracellular matrix. Vascular endothelial growth factor acts as a major factor for angiogenesis and has a direct impact on granulation tissue and the wound healing process (Tonnesen et al., 2000; Bao et al., 2009; Eming and Krieg, 2006). Nevertheless, the healing of the tissue requires the resolution of the inflammation process, which, if prolonged, can impair the healing process and result in a non-healing wound (Eming et al., 2007; Roy, 2010).

Although significant progress has been made with respect to wound care, chronic and non-healing wounds remain a major clinical problem, especially in diabetic patients as well as those with vascular disease or aging. In most of these cases, standard wound dressings merely act as passive isolating barriers of the organism to the environment that do not actively promote the cellular and molecular mechanisms that must be orchestrated for successful wound healing (Frykberg and Banks, 2015). As a result, there is increasing interest for the development of biomaterials that can “fine-tune” the wound microenvironment so as to help in fibroblast migration, angiogenesis and the right organisation of extracellular matrix (Riaz et al., 2025).

As biocompatible, biodegradable materials with similar structure to native extracellular matrix (ECM), natural polymer-derived biomaterials have been widely studied for wound healing. Nanocellulose has become an attractive material among those available materials by virtue of high specific surface area, mechanical rigidity, water retention capacity and cell adhesion and migration properties (Zarepour et al., 2024; Kaur et al., 2021; Ong et al., 2023). A particularly relevant aspect is that nanocellulose can be obtained from renewable sources (e.g., agricultural waste), an advantage not only in terms of sustainability, but also in scalability and material sourcing (Mateo et al., 2021; Ansari et al., 2024; Ghamari et al., 2025).

Previous studies have reported that nanocellulose-based materials are generally non-toxic and can support fibroblast viability and migration, which are critical characteristics for effective wound healing (Ong et al., 2023; Rafee et al., 2025; Szustak and Gendaszewska-Darmach, 2021). In addition, nanocellulose dressings have been shown to maintain a moist wound environment and to promote granulation tissue formation and re-epithelialisation in both preclinical and clinical studies (Breijaert et al., 2025; Pasaribu et al., 2024). However, the inflammatory response elicited by biomaterials remains an important consideration, as abnormally elevated expression of pro-inflammatory cytokines, including interleukin-6 (IL-6), may negatively affect the wound healing process (Johnson et al., 2020; Lin et al., 2003).

The aim of this study was to investigate wheat straw, a plentiful agricultural waste, as a biomaterial for cutaneous wound healing. We characterised the structural and physicochemical properties of the extracted nanocellulose and assessed its effects on fibroblast viability, migration, and the expression of genes and proteins associated with extracellular matrix production and angiogenesis *in vitro*. In addition, we evaluated the impact of nanocellulose treatment on macroscopic wound closure and histological features of healing using a rat full-thickness skin wound model. By employing this combined *in vitro* and *in vivo* approach, we sought to determine whether nanocellulose could enhance wound healing while avoiding excessive stimulation of inflammatory responses.

## Materials and methods

### Preparation of cellulose from wheat straw

A wheat straw agricultural waste was identified to isolate cellulose. The raw material was washed well with the help of distilled water in order to get rid from surface impurities followed by dried in the air at room temperature. Mechanical powdering was performed on the dried materials. To remove non-cellulosic materials, the wheat straw powder was treated with a 2% (w/v) sodium hydroxide solution and stirred at 80 °C for 4 h. The treated material was then rinsed several times with deionised water until a neutral pH of 7 was obtained, filtered and dried under air. Subsequent bleaching was performed to remove remaining lignin and phenolic content by immersing the material in sodium hypochlorite solution at room temperature for 48 h. The samples were then filtered and extensively washed using distilled water until neutral pH was achieved, and finally dried and milled to produce purified cellulose powder. All reagents used were of analytical grade, and all procedures were performed under standard laboratory safety conditions.

### Extraction of nanocellulose

Nanocellulose was extracted from the purified cellulose using the sulfuric acid hydrolysis method. In detail, 10 g of cellulose powder was mixed with 100 mL of 30% sulfuric acid, and the mixture was vigorously stirred at a constant temperature of 30 °C to facilitate the swelling of the cellulose powder before the hydrolysis process. After the hydrolysis process, the reaction was stopped, and the solution was washed with distilled water to remove the excess acid. Washing was conducted until the pH of the solution was almost neutral. The obtained nanocellulose suspension was subjected to ultrasonication with a probe-type sonicator (SONOPULS HD 2070, Bandelin Electronic, Germany) for 80 min in order to enhance dispersion and induce fibrillation. The sonicated nanocellulose was then used for material characterisation and membrane preparation.

### Physicochemical characterisation of wheat straw–derived nanocellulose

#### Field-emission scanning electron microscopy (FESEM) analysis

The surface morphology and nanoscale structural features of the prepared nanocellulose were examined using field-emission scanning electron microscopy (FESEM; ZEISS SIGMA VP, Carl Zeiss, Germany). Before FESEM examination, samples were fixed on aluminum stubs and sputter-coated with a thin layer of gold to prevent charging effects during microscopy. Micrographs were recorded at different magnifications to study the morphology of the particles. Representative micrographs were selected from repeated observations to confirm reproducibility of the morphology.

#### X-ray diffraction (XRD) analysis

The crystalline structural organization of the crystallized nanocellulose was examined by X-ray diffraction using a Shimadzu XRD-7000 diffractometer (Shimadzu Corporation, Japan) with Cu Kα radiation of wavelength 1.54 Å. Diffraction patterns were recorded over an appropriate range of 2θ at room temperature. Crystallite size was determined using the Debye–Scherrer formula, where full width at half maximum (FWHM) of the major diffraction peak was used. Reproducibility of diffraction patterns was checked by repeating measurements.

#### Energy-dispersive X-ray spectroscopy (EDX) analysis

Elemental composition analysis of the nanocellulose samples was performed using energy-dispersive X-ray spectroscopy (EDX) coupled to the FESEM system. Spectra were collected from multiple representative regions of each sample to evaluate elemental distribution and confirm material purity following chemical treatment.

### Fourier transform infrared spectroscopy

Fourier Transform Infrared Spectroscopy (FTIR) was used to confirm the chemical structure of the extracted nanocellulose and also confirm the removal of non-cellulosic compounds following the chemical treatment. The dried powder samples of the nanocellulose material were investigated using an attenuated total reflection Fourier Transform Infrared Spectrometer (ATR-FTIR; Nicolet iS10, Thermo Fisher Scientific, United States) within a range of 4,000–400 cm^−1^ at a spectral resolution of 4 cm^−1^. The experiment was conducted under ambient conditions with the scans averaged for reproducibility.

#### Preparation of nanocellulose membranes

Nanocellulose films were prepared using a solvent casting technique. In general, 4 g of nanocellulose gel was mixed with 20 mL of deionized water and stirred for about 3 h to obtain a homogenous solution. Subsequently, the solution was cast onto a petri dish of 8 cm diameter and placed near a heat source to allow the evaporation of water. Once the nanocellulose films were completely dry, they were carefully removed from the petri dish and stored for future use. Before using the nanocellulose membranes for biological tests, they were aseptically sterilized using UV light for a specified amount of time on both surfaces. Sterilized nanocellulose membranes were stored using sterile equipment and containers.

#### Preparation of nanocellulose-conditioned medium

Nanocellulose used for the *in vitro* experiments was prepared from wheat straw as described above and handled under aseptic conditions to minimise microbial or endotoxin contamination. Nanocellulose membranes were incubated in complete culture medium at a ratio of 1 mg/mL for 24 h under standard buffered cell culture conditions, and the conditioned medium was visually inspected prior to use to exclude turbidity or particulate contamination. The nCM was collected and used immediately for subsequent experiments. Prior to application in cell-based assays, conditioned medium was allowed to equilibrate following membrane incubation; therefore, observed cellular responses reflect exposure to the conditioned environment rather than direct contact with bulk nanocellulose membranes. Control samples consisted of culture medium incubated under identical conditions in the absence of nanocellulose.

### Cell culture

Mouse embryonic fibroblast NIH-3T3 cells were purchased from the American Type Culture Collection (ATCC), Manassas, VA, United States. These cells were used to mimic the process of dermal wound healing *in vitro*. The cells were grown in Dulbecco’s Modified Eagle’s Medium (DMEM) supplemented with fetal bovine serum (FBS) and antibiotics. The cells were grown in a humidified incubator at 37 °C with 5% CO_2_. For using these cells in experiments, they were subcultured regularly at 80% confluence and used at lower passage numbers. The morphology of these cells was regularly checked during culture.

### Cell viability assay

NIH-3T3 fibroblasts were cultured at a density of 1 × 10^4^ cells/well in a 96-well plate and allowed to adhere overnight under standard tissue culture conditions. The cells were then treated with nCM or control conditioned medium for 24 and 48 h. At the end of the treatment period, the cells were analyzed using a microplate reader (ELx800; BioTek Instruments Inc., Winooski, VT, United States), and the viability was expressed as a percentage relative to the control group. For the viability assay, each experiment was done with three biological replicates.

### *In vitro* wound-healing (scratch) assay

The migratory ability of NIH-3T3 fibroblasts was investigated using an *in vitro* scratch wound healing assay. The cells were seeded in 12 wells and allowed to reach confluence. After this, a scratch wound was created using a sterile 200 µL pipette tip. The cells were then allowed to migrate in the presence of control conditioned medium for the experimental wells, whereas control wells received regular medium. Images of the wound area were captured immediately after scratch formation (0 h) and after 24 h of incubation. To ensure experimental consistency, scratches were generated using the same pipette tip under identical conditions, and comparable initial wound widths were verified across all experimental groups at the 0 h time point prior to treatment. Wound closure was quantified using ImageJ software (National Institutes of Health, United States) by measuring the wound area at each time point, and the percentage of wound closure was calculated as (A_0_ − A_t_)/A_0_ × 100, where A_0_ represents the initial wound area at 0 h and A_t_ represents the wound area after 24 h. Image acquisition and analysis were performed using identical settings for all experimental groups. Scratch wound-healing assays were performed in three independent biological experiments, with each experimental condition analysed in triplicate wells for quantitative assessment of wound closure.

### RNA extraction and quantitative real-time PCR

NIH-3T3 fibroblasts were treated with nCM or control conditioned medium, as indicated. Following treatment, total RNA was isolated using a commercial RNA extraction kit according to the manufacturer’s recommendations. RNA quality and concentration were determined spectrophotometrically. First-strand cDNA was synthesised from equal amounts of total RNA using a commercial reverse transcription kit following the manufacturer’s instructions. Quantitative real-time PCR (qRT-PCR) was performed using SYBR Green chemistry in combination with a commercial master mix (PowerUp™ SYBR® Green Master Mix, Applied Biosystems, United States) on a real-time PCR system (StepOnePlus™, Applied Biosystems, Thermo Fisher Scientific, United States). Amplification was carried out using standard cycling conditions, consisting of an initial incubation step followed by 40 amplification cycles. The expression levels of wound-healing–related genes, including Col1a1, Col3a1, Vegfa, and Il6, were analysed using Gapdh as the internal reference gene. These gene-specific primers were designed using published mouse sequence data, and the primers used in our study are listed in the Supplementary Material section of the paper. The gene expression analyses were performed using RNA from three different biological experiments, each of which was analyzed in technical triplicate during qRT-PCR amplification to minimize experimental variation.

### Enzyme-linked immunosorbent assay (ELISA)

The quantification of angiogenic and pro-inflammatory factors in the conditioned media from NIH-3T3 fibroblasts was performed using the enzyme-linked immunosorbent assay (ELISA). NIH-3T3 mouse fibroblasts were seeded in 12-well culture plates and allowed to adhere overnight in standard culture conditions. After this, the cells were treated with nCM or conditioned medium for 24 h. The conditioned media were collected after 24 h and centrifuged at 14,000 x g to remove any cellular debris. These were then frozen at −80 °C before ELISA. ELISA was used to determine the concentration of vascular endothelial growth factor (VEGF) and interleukin-6 (IL-6) in the conditioned media using commercially available ELISA kits following the manufacturer’s instructions. Generally, standards and samples were added to antibody-coated wells in a microplate and then left to stand to facilitate antigen-antibody binding. After this, the unbound materials were removed from the wells using a washing solution. An enzyme-conjugate solution was then added to the wells to bind to the antigen-antibody complexes. Finally, a chromogenic substrate solution was added to the wells to facilitate color development. The reactions were stopped using a stop solution, and the results were read using a microplate reader at 450 nm. ELISA was performed on conditioned media from three different biological experiments. For each experiment, duplicate wells were used to perform the ELISA following the manufacturer’s instructions.

### Experimental animals and ethical approval

Male Sprague–Dawley rats weighing 150–200 g and aged 16–20 weeks were used in this study. Animals were kept under standard environmental conditions (22 °C ± 2 °C) and had free access to food and water. All experimental procedures were carried out in accordance with the guidelines of the Ethical Approval Committee of the Ministry of Higher Education and Scientific Research, University of Anbar, Iraq (Ref. No. 99; Date: 9 January 2025). Before the initiation of the experiment, animals were acclimatized to the experimental conditions. Every precaution was taken to minimize the suffering and distress of the animals throughout the experimental period. Animals showing signs of severe distress, infection, and poor recovery were treated appropriately. Animals were monitored for their general health condition throughout the experimental period. Humane endpoints were implemented to follow the institutional guidelines on animal welfare.

### Wound creation and macroscopic wound assessment

Twenty male Sprague–Dawley rats were randomly assigned to two groups using a simple randomization technique, with each animal considered a unit of randomization. Allocation to treatment groups, either untreated controls or nanocellulose treatment, occurred before the surgical intervention and was done by people who were not involved in outcome measurement. Rats were anaesthetised before wound creation, and the dorsal skin was shaved and disinfected with povidone–iodine solution. A full-thickness linear wound approximately 3 cm in length extending to the myofascial fascia was created on the dorsum of each animal. Nanocellulose membranes were applied immediately after wound induction in the treatment group, whereas control animals received no treatment. Nanocellulose was applied in the form of sterile membranes cut to match the wound dimensions, ensuring complete coverage of the wound surface. A single membrane was applied per wound immediately after injury and maintained in contact with the wound bed under sterile gauze dressing for 3 days without reapplication. Control wounds received identical sterile gauze dressing without nanocellulose to ensure comparable wound protection conditions. No positive comparator dressing was included in the present study, as the objective was to evaluate the intrinsic wound-healing potential of nanocellulose relative to untreated controls. Wounds were covered with sterile gauze and secured using a non-compressive bandage. Macroscopic wound assessment was performed on days 3, 7, and 14 after injury. Digital photographs were obtained using a digital camera (EOS 80D, Canon Inc., Tokyo, Japan) positioned at a fixed distance under standardised lighting conditions, with a measurement scale included for calibration. Image acquisition and wound-area quantification were performed by investigators blinded to group allocation using ImageJ software (National Institutes of Health, United States). Wound closure was expressed as the percentage reduction in wound area relative to baseline. Each animal was considered an independent biological replicate, and percentage wound closure was defined as the primary experimental endpoint. All animals completed the study and were included in the final analysis, with no exclusions or losses observed.

### Histological evaluation

Tissue samples of the skin, including the wound sites and the surrounding tissues, were obtained from the animals on the third, seventh, and fourteenth days following the wounding. The obtained tissues were washed with phosphate-buffered saline solution to remove blood and tissue debris. Fixation of the tissues was conducted using 10% formaldehyde solution at room temperature for 24 h. Dehydration of the fixed tissues was conducted using a series of ethanol solutions of increasing concentrations, ranging from 70% to absolute ethanol. Subsequently, the tissues were cleared using xylene, and paraffin wax was used to embed the tissues. Tissue sections of 4 μm thickness were obtained from the paraffin-embedded tissues using a rotary microtome. Deparaffination of the sections was conducted using xylene, followed by hydration of the sections using a series of alcohol concentrations. Finally, the sections were cleared with distilled water. Routine staining was conducted to assess general tissue repair, and the sections were examined using a microscope. For histological evaluation, three tissue sections were analysed per animal, and five non-overlapping microscopic fields were examined from each section within comparable regions of the wound area. Histological evaluation focused on parameters relevant to cutaneous wound healing, including the extent of inflammatory cell infiltration, granulation tissue formation and organisation, re-epithelialisation, continuity and thickness of the epidermis, dermal architecture, collagen deposition patterns, and the presence of neovascularisation. Representative images were captured at appropriate magnifications to document histological features of tissue regeneration and maturation.

### Statistical analysis

Data are presented as mean ± SD. GraphPad Prism software (V.10) was used for all statistical analyses and graphical work. For experiments involving a single independent variable at a single time point (for example, gene expression and ELISA analyses), one-way analysis of variance (ANOVA) was performed. For experiments involving two independent variables, such as treatment and time (cell viability assays, *in vitro* scratch wound-healing assay, and *in vivo* wound closure analysis), two-way ANOVA was conducted followed by Šídák’s multiple-comparison *post hoc* test. Prior to statistical testing, data distribution was assessed for consistency with parametric assumptions. All statistical tests were two-tailed, and a *p* value of less than 0.05 was considered statistically significant.

## Results

### Structural and physicochemical characterisation of wheat straw-derived nanocellulose

**FIGURE 1:**
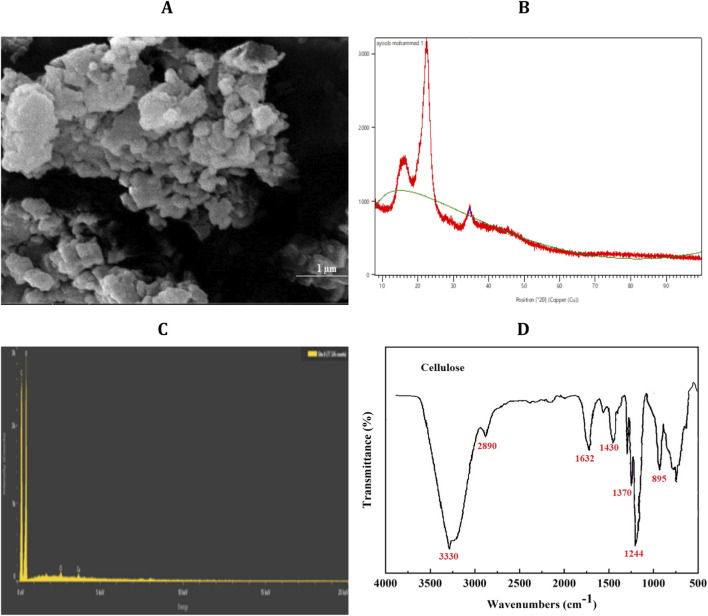
Physicochemical characterisation of wheat straw–derived nanocellulose. **(A)** FESEM micrograph showing the surface morphology and nanoscale aggregation of the prepared nanocellulose, scale bar = 1 µm. **(B)** XRD pattern confirming the crystalline cellulose I structure. **(C)** Energy-dispersive EDX spectrum demonstrating elemental composition and material purity. **(D)** Fourier transform infrared (FTIR) spectrum showing characteristic functional groups of purified nanocellulose.

The physicochemical properties of nanocellulose were characterised using FESEM, XRD, EDX, and FTIR analyses, as presented in Figure 1. FESEM micrographs (Figure 1A) reveal that the nanocellulose consists of loosely aggregated irregular nanoparticles forming fibrillar structures with clearly defined nanoscale domains. Particle size analysis showed that nanocellulose diameters ranged from 32 to 74 nm, with an average size of approximately 53 nm. XRD analysis (Figure 1B) demonstrated distinct diffraction peaks at 2θ values of approximately 15.6°, 22.3°, and 34.6°, corresponding to the (110), (200), and (004) lattice planes of cellulose I. These findings indicate that the native crystalline structure of cellulose was preserved following the extraction process. The average crystallite size was estimated to be approximately 14.9 nm. EDX spectroscopy (Figure 1C) confirmed that the material was predominantly composed of carbon and oxygen, consistent with the elemental composition of cellulose. The absence of detectable inorganic elements further indicates the effectiveness of the purification process in removing non-cellulosic components. FTIR spectroscopy further supported the chemical structure of the isolated nanocellulose (Figure 1D). A broad absorption band observed around 3,330 cm^−1^ corresponds to O–H stretching vibrations associated with intermolecular hydrogen bonding. The absorption band around 2,890 cm^−1^ is attributed to C–H stretching of glucopyranose units. Additional characteristic bands observed at 1,632, 1,430, and 1,244 cm^−1^ correspond to absorbed water bending, CH_2_ bending, and C–O stretching vibrations, respectively. The prominent absorption band at approximately 895 cm^−1^ confirms the presence of β-1,4-glycosidic linkages characteristic of cellulose. Importantly, the absence of absorption bands associated with lignin and hemicellulose indicates efficient removal of non-cellulosic constituents during chemical treatment. Collectively, these findings confirm the successful isolation of chemically purified nanocrystalline cellulose from wheat straw while preserving its structural integrity and characteristic functional groups.

### Nanocellulose exhibits no cytotoxic effect on NIH-3T3 fibroblasts

**FIGURE 2:**
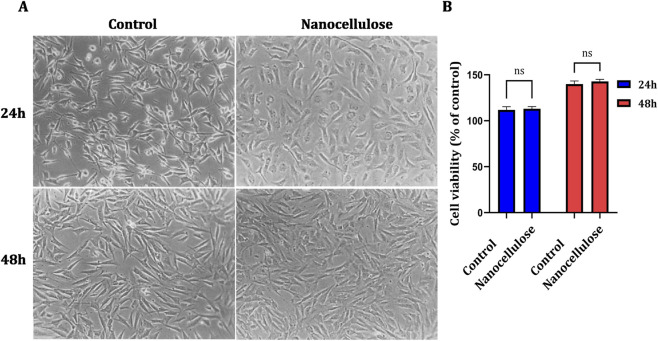
Effect of nanocellulose on NIH-3T3 fibroblast viability. **(A)** Representative phase-contrast micrographs of NIH-3T3 fibroblasts cultured under control conditions or treated with nanocellulose-conditioned medium for 24 h and 48 h, showing preserved cell morphology and confluence, scale bar = 100 µm. **(B)** Quantitative analysis of fibroblast viability at 24 h and 48 h, expressed as a percentage relative to the control group. Data are presented as mean ± SD (n = 3). Statistical analysis was performed using two-way ANOVA followed by Šídák’s multiple comparison test; no significant differences were observed between control and nanocellulose-treated groups (ns, p > 0.05).

NIH-3T3 fibroblasts appeared to be essentially unchanged in morphology and exhibited good attachment to the culture surface after 24 h and 48 h, respectively, as visualized in representative phase-contrast micrographs (Figure 2A) exposed to nanocellulose-conditioned medium compared with control untreated cells. No cytopathic features (cell rounding, detachment, or decrease in confluence) were detected at either time point. These findings were consistent with the results of quantitative cell viability analysis. At 24 h, fibroblast viability in the nanocellulose-treated group did not differ significantly from that of the control group. Similarly, after 48 h of treatment, no significant difference in cell viability was detected between treated and untreated cells (Figure 2B). Two-way ANOVA followed by Šídák’s multiple-comparison test confirmed the absence of statistically significant differences between control and nanocellulose-treated cells at both time points (p > 0.05). Taken together, these results indicate that nanocellulose does not exert cytotoxic effects on NIH-3T3 fibroblasts following either short-term or longer-term exposure.

### Nanocellulose significantly enhances fibroblast migration and wound closure *in vitro*

**FIGURE 3:**
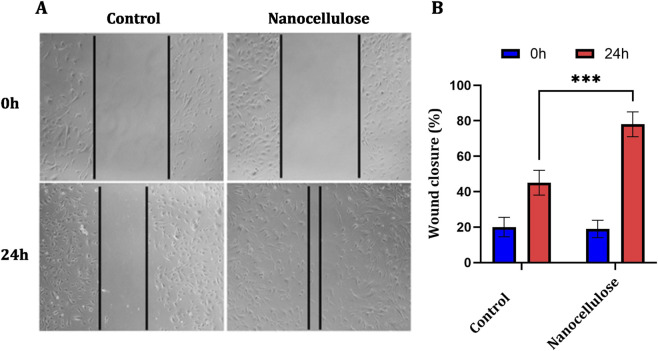
Nanocellulose enhances fibroblast migration and wound closure *in vitro*. **(A)** Representative phase-contrast images of scratch wound assays performed in NIH-3T3 fibroblast monolayers under control conditions or following treatment with nanocellulose-conditioned medium at 0 h and 24 h. Black lines indicate the initial wound boundaries, scale bar = 100 µm. **(B)** Quantitative analysis of wound closure expressed as the percentage of wound closure at 0 h and 24 h. Data are presented as mean ± SD (n = 3). Statistical analysis was performed using two-way ANOVA followed by *post hoc* multiple comparisons. Significant differences between time points and treatment groups are indicated (***p < 0.001), while non-significant differences are denoted as ns.

Scratch assay representative images demonstrated gradual closure of the wound within 24 h for both control and nanocellulose-treated NIH-3T3 fibroblast monolayers (Figure 3A). There was no significant difference in wound width for control versus nanocellulose-treated at the initial time point (0 h), suggesting scratch production was similar across treatments. At 24 h, fibroblasts were noted to have migrated into the wound site in both groups but with a much more pronounced decrease in wound width for nanocellulose-treated cells. These data were supported by quantitative measurements (Figure 3B). In the control group wound contraction dramatically increased at 24 h compared to 0 h, indicating normal fibroblast migration. By comparison, nanocellulose treatment showed a distinctly greater promotion of wound closure over this time. The percent of wound closure for the nanocellulose-treated group was significantly greater than that found in control cells at 24 h. Post hoc multiple-comparison analysis of the two-way ANOVA revealed that in both groups, there was a significant time-dependent improvement in wound closure, with significantly greater effect for nanocellulose-treated group at 24 h (*p* < 0.001). At 0 h, there were no significant differences between control and nanocellulose-treated groups, which shows that the effect was the result of increased fibroblast migration over time of incubation.

### Nanocellulose modulates wound-healing related gene expression and protein secretion in fibroblasts

**FIGURE 4:**
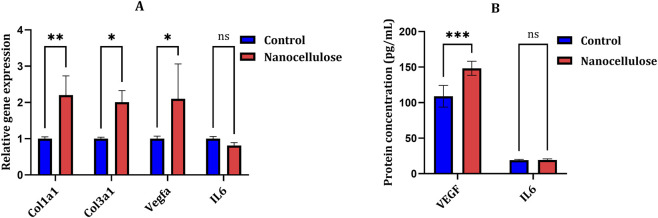
Nanocellulose modulates wound-healing–related gene expression and protein secretion in fibroblasts. **(A)** Relative mRNA expression levels of *Col1a1, Col3a1, Vegfa*, and *IL-6* in NIH-3T3 fibroblasts treated with nanocellulose-conditioned medium for 24 h. Gene expression was normalised to Gapdh and expressed relative to the control group. **(B)** Protein concentrations of VEGF and IL-6 in conditioned media collected after 24 h, as determined by ELISA. Data are presented as mean ± SD (n = 3). Statistical analysis was performed using one-way ANOVA. *p < 0.05; **p < 0.01; ***p < 0.001; ns, not significant.

A significant change of wound-healing–related gene transcription profile was detected in NIH-3T3 fibroblasts when treated with nanocellulose-treated medium (Figure 4A). *Col1a1* expression was significantly higher in nanocellulose-treated cells relative to control cells (*p* = 0.0093), which indicated the activation of transcription for collagen type I. Furthermore, *Col3a1* was found to be markedly upregulated in response to nanocellulose (*p* = 0.0308), indicating early activation of remodelling of the extracellular matrix processes. Among the angiogenesis-related genes, *Vegfa* also displayed a significantly higher expression upon nanocellulose treatment (*p* = 0.0175). In contrast, there was no significant modulation of relative expression of Il6 in nanocellulose treated compared to control cells (*p* = 0.9675), that is, exposure to nanocellulose did not trigger an inflammatory transcriptional response. Correspondingly, the ELISA analysis from conditioned media confirmed our gene expression data at protein level (Figure 4B). VEGF protein secretion was significantly higher in fibroblasts treated with nanocellulose compared with control cells (*p* = 0.0007), indicating enhanced release of an angiogenic factor. Conversely, IL-6 protein levels were not significantly different between the two groups (*p* = 0.9657), confirming the absence of a pro-inflammatory response at the protein level. Inflammatory response was evaluated by analysing Il6 gene expression and IL-6 protein secretion following exposure to nanocellulose-conditioned medium at 24 and 48 h. IL-6 concentrations were quantified using ELISA within the detection range specified by the manufacturer, and values obtained in nanocellulose-treated samples remained comparable to control conditions at all analysed time points. Taken together, these results indicate that nanocellulose promotes fibroblast transcriptional activation and protein secretion associated with extracellular matrix formation and angiogenesis, without stimulating inflammatory signalling pathways.

### Nanocellulose treatment improves macroscopic wound healing outcomes

**FIGURE 5:**
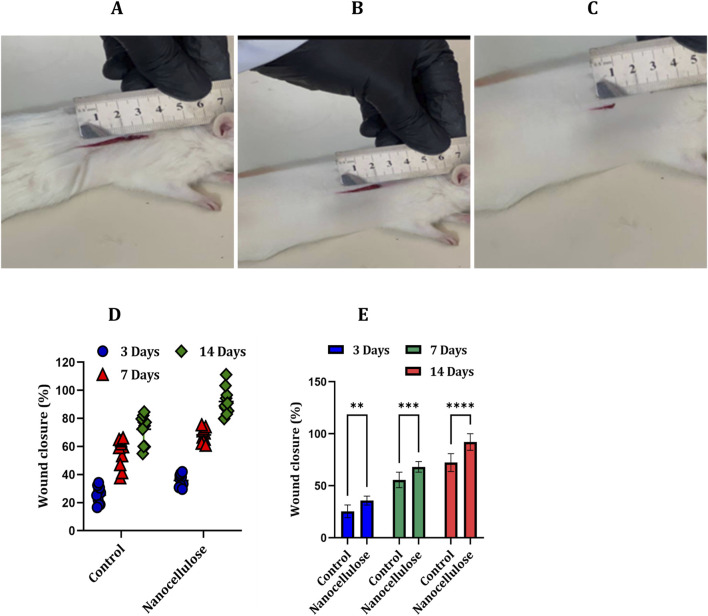
Nanocellulose accelerates macroscopic wound closure *in vivo*. Representative photographs of dorsal full-thickness skin wounds showing healing progression at day 3 **(A)**, day 7 **(B)**, and day 14 **(C)** post-injury. A measurement ruler was included in each image to provide spatial reference for wound size assessment. **(D)** Individual wound-closure values for each animal expressed as percentage closure relative to the initial wound area (n = 10 animals per group). **(E)** Quantitative comparison of wound closure between control and nanocellulose-treated groups presented as mean ± SD. Statistical analysis was performed using two-way ANOVA followed by Šídák’s multiple comparison test (**p < 0.01; ***p < 0.001; ****p < 0.0001).

Macroscopic evaluation of wound healing demonstrated a gradual reduction in wound size in both experimental groups over the experimental period; however, animals treated with nanocellulose exhibited visibly faster wound contraction compared with untreated controls. Representative images obtained at days 3, 7, and 14 post-injury show a more pronounced decrease in wound area in the nanocellulose-treated group, characterised by earlier narrowing of wound margins and improved surface closure (Figures 5A–C). Quantitative analysis of wound closure supported these visual observations, revealing a greater percentage reduction in wound area in nanocellulose-treated animals compared with control wounds at all examined time points (Figures 5D,E). Presentation of individual animal data demonstrated a consistent pattern of enhanced wound closure among treated animals, indicating that the observed effect was reproducible across the experimental cohort rather than driven by isolated responses. Statistical comparison further confirmed significantly improved wound closure following nanocellulose treatment, with differences becoming more pronounced during the later stages of healing, particularly at days 7 and 14. These findings suggest that nanocellulose application promotes accelerated progression of wound repair towards tissue regeneration and remodelling.

### Nanocellulose accelerates tissue regeneration and histological maturation during cutaneous wound healing

**FIGURE 6:**
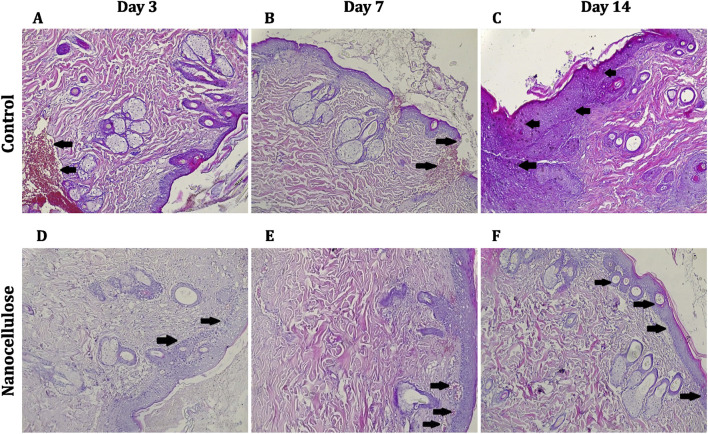
Histological evaluation of wound healing following nanocellulose treatment. Representative haematoxylin and eosin (H&E)–stained sections of wound tissues collected at days 3, 7, and 14 post-injury, scale bar = 200 µm. Panels **(A–C)** represent untreated control wounds at days 3, 7, and 14, respectively, while panels **(D–F)** represent nanocellulose-treated wounds at the corresponding time points. At day 3, control wounds **(A)** show marked inflammatory cell infiltration and poorly organised granulation tissue (black arrows), whereas nanocellulose-treated wounds **(D)** exhibit reduced inflammation and early tissue organisation. At day 7, control wounds **(B)** display disrupted epidermal architecture and persistent inflammation (black arrows), while treated wounds **(E)** demonstrate enhanced granulation tissue formation, increased neovascularisation, and improved collagen organisation (black arrows). At day 14, control wounds **(C)** show incomplete re-epithelialisation, residual inflammation, and abnormal epidermal thickening (black arrows), whereas nanocellulose-treated wounds **(F)** reveal a continuous mature epidermis, organised dermal structure, minimal inflammation, and regeneration of skin appendages, including hair follicles (black arrows).

Sections of wound tissue stained with haematoxylin and eosin (H&E) were examined to evaluate the effects of nanocellulose treatment on the inflammatory response, re-epithelialisation, granulation tissue formation, angiogenesis, and dermal remodelling at days 3, 7, and 14 post-injury (Figure 6).

On day 3, wounds from the untreated control (Figure 6A) were characterized by extensive infiltration of inflammatory cells in the dermis and by poorly organized granulation tissue and little epithelial regeneration at wound edges, indicative of early inflammation phase healing. In contrast, in wounds treated with nanocellulose (Figure 6D), the inflammatory cell infiltration was limited and there was early organization of granulation tissue, suggesting suppression of the inflammation phase and an accelerated transition into subsequent phases such as proliferation.

On day 7, control skin wounds (Figure 6B) still contained intense inflammatory infiltrate and disorganized epithelial structure indicative of delayed re-epithelization and tissue remodeling. In contrast to this, nanocellulose treated wounds (Figure 6E) showed well-formed granulation tissue of increased extent, with neovascular structures and enhanced collagen deposition along with more orderly dermal components were seen indicating active angiogenesis and robust fibroproliferative activity.

By day 14, the control wounds (Figure 6C) histological sections were showing incomplete and uneven epidermal integrity, continuing presence of inflammatory cells and patchy thickening of epidermis which suggests that tissue remodelling was delayed in these wounds resulting to poor wound maturation. In contrast, wounds treated with nanocellulose (Figure 6F) had a cohesive and well-stratified epidermis, organized collagen fibers within the dermis, minimal inflammatory infiltration, and regeneration of skin appendages including hair follicles. These characterstics are similar to that of the post-remodel tissue and almost fully recovered wounds.

Taken together, these histological findings indicate that nanocellulose treatment improves both the quality and progression of cutaneous wound healing by modulating early inflammation, promoting angiogenesis and collagen organisation, and facilitating complete re-epithelialisation and tissue regeneration. These tissue-level observations are in agreement with the enhanced fibroblast migration, increased expression of wound-healing–related genes, elevated VEGF protein secretion, and accelerated macroscopic wound closure observed in earlier analyses.

## Discussion

In the present study, we demonstrate that wheat straw–derived nanocellulose can facilitate cutaneous wound healing by promoting fibroblast migration, extracellular matrix expression, and angiogenesis, while not inducing excessive inflammatory responses. By combining material characterisation with *in vitro* fibroblast assays, an *in vivo* rat wound model, and histological analysis, we show that nanocellulose functions as a biocompatible biomaterial capable of modulating key biological processes involved in skin repair.

The nanocellulose prepared in the present study preserved the native crystalline cellulose I structure and nanoscale particle size, similar to those of previously reported plant-based acid hydrolysed (Trache et al., 2020; Klemm et al., 2011). It is imperative to retain the cellulose crystallinity and nanoscale morphology; which are related to surface area, water-retention capacity and interaction with cells and extracellular matrix components (Moon et al., 2011). Moreover, the elemental composition analysis demonstrated that non-cellulosic impurities were effectively removed from the extracted nanocellulose thus making them compatible for biological applications.

Biocompatibility is a fundamental requirement for materials intended for wound healing applications. In the present study, *in vitro* analysis showed that NIH-3T3 fibroblasts maintained normal morphology and exhibited no meaningful reduction in viability following exposure to nanocellulose-conditioned medium (nCM) over the tested time points. These findings are in agreement with previous reports demonstrating minimal cytotoxicity of nanocellulose-based materials in fibroblast and keratinocyte cultures (Barja, 2021; Samyn et al., 2023; Chandana et al., 2022). The absence of cytotoxic effects suggests that nanocellulose does not release harmful by-products during incubation and can therefore be safely introduced to mammalian cells.

Migration of fibroblasts into the wound site is a critical component of the proliferative phase of healing, as it enables granulation tissue formation and wound contraction. In the scratch wound-healing assay, nanocellulose treatment significantly promoted fibroblast migration compared with control conditions. Similar pro-migratory effects of nanocellulose scaffolds have been reported previously and have been attributed to favourable surface properties, hydration capacity, and indirect modulation of cell–matrix interactions (Jacob et al., 2022; Paliwal and Lather, 2024). The enhanced fibroblast migration observed in the present study provides a functional explanation for the accelerated wound closure detected *in vivo*. The biological effects observed following treatment with nanocellulose-conditioned medium may result from soluble components released during conditioning, physicochemical modification of the culture medium, or residual nanoscale material. As the present study was not designed to distinguish between these mechanisms, further studies using particle-free filtrates or physicochemical characterisation of conditioned media are required.

Recent studies have increasingly demonstrated the effectiveness of nanocellulose-based biomaterials in promoting cutaneous wound repair through modulation of fibroblast activity and extracellular matrix organisation. In particular, bacterial nanocellulose and plant-derived nanocellulose scaffolds have been reported to enhance fibroblast migration, support cell adhesion, and accelerate wound closure by providing a hydrated microenvironment that mimics native extracellular matrix architecture. The pro-migratory effects observed in the present study are consistent with these previous findings, suggesting that enhancement of fibroblast dynamics represents a common regenerative mechanism associated with nanocellulose materials regardless of their biological source. Importantly, the present work extends these observations by demonstrating that nanocellulose derived from wheat straw agricultural waste can achieve comparable biological responses while offering a sustainable and low-cost alternative for wound-healing applications (Aderibigbe, 2024).

At the molecular level study, application of nanocellulose was shown to upregulate *Col1a1* and *Col3a1* which indicates activation of dermal matrix synthesis (collagen) pathways. Type III Col is essential for the early phase of wound healing, and it acts as a provisional matrix that is substituted for type I Coll during tissue remodelling (Diller and Tabor, 2023). Moreover, higher *Vegfa* expression together with increased VEGF protein secretion indicates the ability of nanocellulose to promote angiogenic signalling, which is important for oxygen and nutrient supply to regenerating tissue (Shams et al., 2022; Bao et al., 2009; Goswami et al., 2022). Notably, no induction of *Il6* expression or IL-6 protein release was observed, suggesting that nanocellulose does not elicit an excessive or prolonged pro-inflammatory response under the experimental conditions used, which is a crucial consideration for biomaterials designed for wound healing applications (Sugawara et al., 2001; Lin et al., 2003).

Furthermore, the *in vivo* rat wound model indicated that nanocellulose could promote positive effects on tissue repair. Macroscopically, wounds healed faster in animals treated with nanocellulose. The histological analysis demonstrated additional desirable results of nanocellulose relative to the untreated control, including a decrease in inflammatory cell infiltration, well-organised granulation tissue formation, enhanced angiogenesis and more progressive re-epithelialisation. On day 14, the nanocellulose-treated wounds demonstrated the rebuilt epidermal layer of skin, well organised dermal collagen fibres and regeneration of skin appendages indicating proper tissue remodeling and maturation (Singer, 2022; Gushiken et al., 2021; Zeng et al., 2018). These histological observations are in agreement with the *in vitro* findings and provide a consistent biological rationale by which nanocellulose is promoting fibroblast-driven repair. Recent advances in nanocellulose-based biomaterials have highlighted the growing interest in plant-derived and biomass-sourced cellulose as sustainable alternatives for tissue engineering and wound-healing applications. Such materials have demonstrated comparable biological performance to conventional nanocellulose systems while improving material accessibility and environmental sustainability (Patel et al., 2022).

The wound healing and inflammation-promoting effects of nanocellulose may be related to its physicochemical characteristics such as high water-binding capacity and structural resemblance with the native extracellular matrix. Studies have demonstrated that biomaterials that maintain a moist wound environment and enhance cell adhesion promote the transition from inflammation to proliferation (Tan and Dosan, 2019; Nuutila and Eriksson, 2021). Furthermore, the application of wheat straw as a cellulose source suggests using agricultural waste as a sustainable and renewable resources for medical purposes, which coincides with the increasing attention paid to environmentally friendly and sustainable biomaterials (Qureshi et al., 2024; Ren et al., 2025). The potential for foreign body reactions and material persistence should also be considered for biomaterials applied to wound sites. In the present study, histological analysis did not indicate excessive inflammatory infiltration or fibrotic encapsulation, suggesting favourable tissue compatibility. Although nanocellulose is generally regarded as biologically inert, its biodegradation and clearance within the wound environment require further investigation. In addition, as the material is derived from wheat straw, potential hypersensitivity responses related to residual plant components cannot be entirely excluded and warrant evaluation in future studies. Although additional physicochemical characterisation such as rheological behaviour, fibre size distribution, swelling properties, and post-sterilisation stability may further support material optimisation, the present study focused primarily on evaluating the biological performance of nanocellulose in wound-healing applications. Future investigations will address these parameters to provide a more comprehensive physicochemical assessment.

Nevertheless, the present study has some limitations. Although IL-6 levels were investigated as an indicator of inflammatory response, a broader analysis incorporating additional inflammatory mediators and immune cell populations would provide a more comprehensive understanding of the immunological mechanisms involved in nanocellulose-mediated wound healing. In addition, while histological observations suggested enhanced angiogenic activity within nanocellulose-treated wounds, objective quantification of vascular density was not performed, as haematoxylin and eosin staining does not permit reliable identification of endothelial structures required for accurate microvessel analysis. Future studies employing endothelial-specific markers, such as CD31 or von Willebrand factor, would enable more precise evaluation of neovascularisation. Furthermore, assessment of long-term healing outcomes, including biomechanical properties such as tensile strength of the regenerated tissue, as well as direct comparison with clinically established wound dressings in translational models, would further strengthen the clinical relevance of the findings.

In summary, the present study demonstrates that wheat straw–derived nanocellulose enhances cutaneous wound repair by promoting fibroblast migration, collagen deposition, and angiogenesis, while inducing minimal inflammatory activation. The combined *in vitro*, *in vivo*, and histological results consistently support the potential of nanocellulose as a sustainable and biologically functional biomaterial for wound healing applications.

## Data Availability

The raw data supporting the conclusions of this article will be made available by the authors, without undue reservation.

## References

[B1] Aderibigbe B. A. (2024). Application of nanocellulose for wound dressings. Nanocellulose, 193–219. 10.1002/9781394172825.ch8

[B2] Ansari M. M. Heo Y. Do K. Ghosh M. Son Y. O. (2024). Nanocellulose derived from agricultural biowaste by-products–sustainable synthesis, biocompatibility, biomedical applications, and future perspectives: a review. Carbohydr. Polym. Technol. Appl. 8, 100529. 10.1016/j.carpta.2024.100529

[B3] Bainbridge P. (2013). Wound healing and the role of fibroblasts. J. Wound Care 22 (8), 407–410. 10.12968/jowc.2013.22.8.407 23924840

[B4] Bao P. Kodra A. Tomic-Canic M. Golinko M. S. Ehrlich H. P. Brem H. (2009). The role of vascular endothelial growth factor in wound healing. J. Surg. Res. 153 (2), 347–358. 10.1016/j.jss.2008.04.023 19027922 PMC2728016

[B5] Barja F. (2021). Bacterial nanocellulose production and biomedical applications. J. Biomed. Res. 35 (4), 310–317. 10.7555/JBR.35.20210036 34253695 PMC8383174

[B6] Breijaert T. C. Fontes M. Fernandes P. d. A. Barud H. d. S. Ribeiro S. J. Seisenbaeva G. A. (2025). Functionalization of bacterial nanocellulose-based wound dressing for increased drug retention. Carbohydr. Polym. Technol. Appl. 10, 100756. 10.1016/j.carpta.2025.100756

[B7] Chandana A. Mallick S. P. Dikshit P. K. Singh B. N. Sahi A. K. (2022). Recent developments in bacterial nanocellulose production and its biomedical applications. J. Polym. Environ. 30 (10), 4040–4067. 10.1007/s10924-022-02507-0

[B8] Cialdai F. Risaliti C. Monici M. (2022). Role of fibroblasts in wound healing and tissue remodeling on Earth and in space. Front. Bioeng. Biotechnol. 10, 958381. 10.3389/fbioe.2022.958381 36267456 PMC9578548

[B9] Diller R. B. Tabor A. J. (2023). The extracellular matrix (ECM) and wound healing: a review. Res. Adv. Microbiol. Biotechnol. 8, 95–117. 10.9734/bpi/ramb/v8/8007A

[B10] Eming S. A. Krieg T. (2006). Molecular mechanisms of VEGF-A action during tissue repair. J. Investigative Dermatology Symposium Proc. 11 (1), 79–86. 10.1038/sj.jidsymp.5650016 17069014

[B11] Eming S. A. Krieg T. Davidson J. M. (2007). Inflammation in wound repair: molecular and cellular mechanisms. J. Invest Dermatol 127 (3), 514–525. 10.1038/sj.jid.5700701 17299434

[B12] Frykberg R. G. Banks J. (2015). Challenges in the treatment of chronic wounds. Adv. Wound Care (New Rochelle) 4 (9), 560–582. 10.1089/wound.2015.0635 26339534 PMC4528992

[B13] Ghamari M. Suvish Hwang See C. Yu H. Anitha T. Balamurugan V. T. (2025). Nanocellulose extraction from biomass waste: unlocking sustainable pathways for biomedical applications. Chem. Rec. 25 (5), e202400249. 10.1002/tcr.202400249 40035542 PMC12067182

[B14] Goswami A. G. Basu S. Huda F. Pant J. Ghosh Kar A. Banerjee T. (2022). An appraisal of vascular endothelial growth factor (VEGF): the dynamic molecule of wound healing and its current clinical applications. Growth Factors 40 (3-4), 73–88. 10.1080/08977194.2022.2074843 35584274

[B15] Gushiken L. F. Beserra F. P. Bastos J. K. Jackson C. J. Pellizzon C. H. (2021). Cutaneous wound healing: an update from physiopathology to current therapies. Life 11, 665. 10.3390/life11070665 34357037 PMC8307436

[B16] Jacob S. Reshmy R. Antony S. Madhavan A. Sindhu R. Kumar Awasthi M. (2022). Nanocellulose in tissue engineering and bioremediation: mechanism of action. Bioengineered 13 (5), 12823–12833. 10.1080/21655979.2022.2074739 35609323 PMC9275936

[B17] Johnson B. Z. Stevenson A. W. Prêle C. M. Fear M. W. Wood F. M. (2020). The role of IL-6 in skin fibrosis and cutaneous wound healing. Biomedicines 8 (5), 101. 10.3390/biomedicines8050101 32365896 PMC7277690

[B18] Kaur P. Sharma N. Munagala M. Rajkhowa R. Aallardyce B. Shastri Y. (2021). Nanocellulose: resources, physio-chemical properties, current uses and future applications. Front. Nanotechnol. 3, 747329. 10.3389/fnano.2021.747329

[B19] Klemm D. Kramer F. Moritz S. Lindström T. Ankerfors M. Gray D. (2011). Nanocelluloses: a new family of nature-based materials. Angew. Chem. Int. Ed. 50 (24), 5438–5466. 10.1002/anie.201001273 21598362

[B20] Lin Z.-Q. Kondo T. Ishida Y. Takayasu T. Mukaida N. (2003). Essential involvement of IL-6 in the skin wound-healing process as evidenced by delayed wound healing in IL-6-deficient mice. J. Leukoc. Biol. 73 (6), 713–721. 10.1189/jlb.0802397 12773503

[B21] Mateo S. Peinado S. Morillas-Gutiérrez F. La Rubia M. D. Moya A. J. (2021). Nanocellulose from agricultural wastes: products and applications—a review. Processes 9, 1594. 10.3390/pr9091594

[B22] Moon R. Martini A. Nairn J. Simonsen J. Youngblood J. (2011). ChemInform abstract: cellulose nanomaterials review: structure, properties and nanocomposites. Chem. Soc. Reviews 40, 3941–3994. 10.1039/c0cs00108b 21566801

[B23] Nuutila K. Eriksson E. (2021). Moist wound healing with commonly available dressings. Adv. Wound Care (New Rochelle) 10 (12), 685–698. 10.1089/wound.2020.1232 32870777 PMC8568799

[B24] Ong X. R. Chen A. X. Li N. Yang Y. Y. Luo H. K. (2023). Nanocellulose: recent advances toward biomedical applications. Small Sci. 3 (2), 2200076. 10.1002/smsc.202200076 40213493 PMC11935994

[B25] Paliwal S. Lather A. (2024). Application of nanocellulose as implant and grafting materials. Nanocellulose, 335–358.

[B26] Pasaribu K. M. Mahendra I. P. Karina M. Masruchin N. Sholeha N. A. Gea S. (2024). A review: current trends and future perspectives of bacterial nanocellulose-based wound dressings. Int. J. Biol. Macromol. 279, 135602. 10.1016/j.ijbiomac.2024.135602 39276891

[B27] Patel K. Shaikh J. Khan T. (2022). “Advances in nanocellulose for wound healing applications,” in Handbook of nanocelluloses: classification, properties, fabrication, and emerging applications. Editor Barhoum A. (Cham: Springer International Publishing), 677–708.

[B28] Qureshi S. S. Nizamuddin S. Xu J. Vancov T. Chen C. (2024). Cellulose nanocrystals from agriculture and forestry biomass: synthesis methods, characterization and industrial applications. Environ. Sci. Pollut. Res. 31 (49), 58745–58778. 10.1007/s11356-024-35127-3 39340607 PMC11513767

[B29] Rafee S. M. I. A. Alim M. S. U. Alam S. Salem K. S. (2025). Nanocellulose polymorphs for biomedical applications: recent advances, prospects and challenges - a review. Carbohydr. Polym. Technol. Appl. 11, 100916. 10.1016/j.carpta.2025.100916

[B30] Ren M. Gao Y. Liu F. Kong Q. Sang H. (2025). From waste to wonder: biomass-derived nanocellulose and lignin-based nanomaterials in biomedical applications. Int. J. Biol. Macromol. 307, 142373. 10.1016/j.ijbiomac.2025.142373 40122417

[B31] Riaz S. Waheed H. Ahmad F. Khan M. I. Shanableh A. (2025). Natural and synthetic biomaterials, structural matrices-based wound dressings: key properties, material correlation, and adaptability. Regenes. Repair Rehabil. 1 (4), 47–65. 10.1016/j.rerere.2025.10.004

[B32] Roy S. (2010). “Resolution of inflammation in wound healing: significance of dead cell clearance,” in Advances in wound care: volume 1 (New Rochelle: Mary Ann Liebert, Inc.), 253–258.

[B33] Samyn P. Meftahi A. Geravand S. A. Heravi M. E. M. Najarzadeh H. Sabery M. S. K. (2023). Opportunities for bacterial nanocellulose in biomedical applications: review on biosynthesis, modification and challenges. Int. J. Biol. Macromol. 231, 123316. 10.1016/j.ijbiomac.2023.123316 36682647

[B34] Shams F. Moravvej H. Hosseinzadeh S. Mostafavi E. Bayat H. Kazemi B. (2022). Overexpression of VEGF in dermal fibroblast cells accelerates the angiogenesis and wound healing function: *in vitro* and *in vivo* studies. Sci. Rep. 12 (1), 18529. 10.1038/s41598-022-23304-8 36323953 PMC9630276

[B35] Singer A. J. (2022). Healing mechanisms in cutaneous wounds: tipping the balance. Tissue Eng. Part B Rev. 28 (5), 1151–1167. 10.1089/ten.TEB.2021.0114 34915757 PMC9587785

[B36] Sugawara T. Gallucci R. M. Simeonova P. P. Luster M. I. (2001). Regulation and role of interleukin 6 in wounded human epithelial keratinocytes. Cytokine 15 (6), 328–336. 10.1006/cyto.2001.0946 11594800

[B37] Szustak M. Gendaszewska-Darmach E. (2021). Nanocellulose-based scaffolds for chondrogenic differentiation and expansion. Front. Bioeng. Biotechnol. 9, 2021. 10.3389/fbioe.2021.736213 34485266 PMC8415884

[B38] Talbott H. E. Mascharak S. Griffin M. Wan D. C. Longaker M. T. (2022). Wound healing, fibroblast heterogeneity, and fibrosis. Cell Stem Cell 29 (8), 1161–1180. 10.1016/j.stem.2022.07.006 35931028 PMC9357250

[B39] Tan S. T. Dosan R. (2019). Lessons from epithelialization: the reason behind moist wound environment. Open Dermatology J. 13, 34–40. 10.2174/1874372201913010034

[B40] Tonnesen M. G. Feng X. Clark R. A. (2000). Angiogenesis in wound healing. J. Investig. Dermatol Symp. Proc. 5 (1), 40–46. 10.1046/j.1087-0024.2000.00014.x 11147674

[B41] Trache D. Tarchoun A. F. Derradji M. Hamidon T. S. Masruchin N. Brosse N. (2020). Nanocellulose: from fundamentals to advanced applications. Front. Chem. 8, 392. 10.3389/fchem.2020.00392 32435633 PMC7218176

[B42] Zarepour A. Gok B. Budama-Kilinc Y. Khosravi A. Iravani S. Zarrabi A. (2024). Bacterial nanocelluloses as sustainable biomaterials for advanced wound healing and dressings. J. Mater. Chem. B 12 (48), 12489–12507. 10.1039/d4tb01024h 39533945

[B43] Zeng R. Lin C. Lin Z. Chen H. Lu W. Lin C. (2018). Approaches to cutaneous wound healing: basics and future directions. Cell Tissue Res. 374 (2), 217–232. 10.1007/s00441-018-2830-1 29637308

