# Supplementary material for: Histological and biological evaluation of wheat straw–derived nanocellulose in a rat model of cutaneous wound healing
Source: Front Bioeng Biotechnol. 2026 Apr 1;14:1800416. doi: 10.3389/fbioe.2026.1800416 (PMC13079295; doi:10.3389/fbioe.2026.1800416)
Supplement: Supplementary file 1 [file Table1.docx]

**Supplementary Table S1. Primer sequences used for quantitative real-time PCR**

| **Gene** | **Forward primer (5′→3′)** | **Reverse primer (5′→3′)** |
| --- | --- | --- |
| **Col1a1** | GAGCGGAGAGTACTGGATCG | GCTTCTTTTCCTTGGGGTTC |
| **Col3a1** | CAGGGAATCCAGGTGATGCT | GGGACCAATGTCATAGGGTG |
| **Vegfa** | GCACATAGAGAGAATGAGCTTCC | CTCCGCTCTGAACAAGGCT |
| **Il6** | TAGTCCTTCCTACCCCAATTTCC | TTGGTCCTTAGCCACTCCTTC |
| **Gapdh** | AGGTCGGTGTGAACGGATTTG | TGTAGACCATGTAGTTGAGGTCA |
